# Diagnosis and treatment of acute urogenital and genitalia tract traumas: 10-year clinical experience

**DOI:** 10.12669/pjms.314.6116

**Published:** 2015

**Authors:** Qingsong Zou, Qiang Fu

**Affiliations:** 1 Qingsong Zou, MD. Department of Urology, the Affiliated Sixth People’s Hospital of Shanghai Jiaotong University, Shanghai 200233, China; 2 Qiang Fu, MD. Department of Urology, the Affiliated Sixth People’s Hospital of Shanghai Jiaotong University, Shanghai 200233, China

**Keywords:** Urogenital trauma, Genitalia tract, Kidney, Ureter, Bladder, Urethra

## Abstract

**Objective:**

To report our 10-year diagnosis and treatment experience of acute urogenital and genitalia tract traumas and outline the management of the traumatic injury.

**Methods:**

We reviewed the diagnoses and treatments of 208 cases of acute kidney, ureter, bladder, urethra, or male genitalia injuries in our department between March 2002 and March 2012. The patient data including general information, injury position and mechanism, diagnosis and treatment, the follow-up information was analyzed and summarized.

**Results:**

Of 62 patients with renal injury examined by ultrasound and computed tomography (CT) examination, 45 were treated conservatively, 9 with superselective arterial embolization, and 8 with nephrectomy. Intravenous pyelogram (IVP) was conducted in two patients with ureteral injury, one was treated with cystoscopic ureteral catheterization and the other with ureteric reimplantation. Bladder injury (6 patients) confirmed with a waterflood susceptibility test combined with CT scans underwent laparotomy and the bladder suturing was done. Of 92 patients with urethral injury, 6 were treated with a nonoperative approach (indwelling catheter), 18 with urethral realignment, 35 with cystoscopic urethral realignment, 29 with end-to-end anastomotic urethroplasty, and 4 with urethral repairmen. Of the 24 cases with penile injuries, 1 underwent conservative treatment, 8 were treated with debridement and suture ligation, and 15 were managed with suture repair of the penis white membrane. Of the 24 cases with penile injuries, 1 underwent conservative treatment, 8 were treated with debridement and suture ligation, and 15 were managed with suture repair of the penis white membrane. During the follow-up period, 62 patients with renal injury had normal renal function. Neither of the two patients with ureteral injury developed hydronephrosis. Twenty-nine patients with urethral injury suffered from urethral structure. All patients with vesical or genital injury recovered.

**Conclusions:**

Urethra and kidney injuries are the most common acute urogenital system traumas. Superselective arterial embolization can effectively cease bleeding and maximally protect renal function and ureterorenoscopic realignment is an easily operative and minimally invasive technique in the treatment of urethral injuries. As diagnosis and treatment techniques continue to evolve, minimally invasive procedures should be widely used in acute urogenital trauma.

## INTRODUCTION

With economic development, traffic- and industry-related accidents have been increasing. Acute urinary tract trauma, involving many parts of the urinary system, is particularly common in hospitalized patients. Injury to the urinary tract trauma occurs in 10% of abdominal trauma.^1^ Kidney injuries are the most common urinary tract trauma while ureteral trauma is rare due to the small size and protected location of the ureter. The main cause of ureteral injuries is iatrogenic trauma.^2^,^3^ Bladder injuries are associated with pelvic fractures.^4^ Urethral may be caused by blunt or penetrating objects, catheterization or surgical measures.^5^ Symptoms vary depending on which part of the urinary tract is involved; thus, diagnosis and treatment methods vary. In this article, we have reviewed the cases of the acute urogenital and genitalia tract traumas and summarized the clinical experience of diagnoses and treatments.

## METHODS

We reviewed the diagnoses and treatments of 208 cases of acute kidney, ureter, bladder, urethra, or male genitalia injuries in our department between March 2002 and March 2012. Among them, sixty-two cases (29.81%) were kidney injuries, two cases (0.96%) were ureteral injury, six patients (2.88%) were bladder injury, 92 cases (44.23%) were urethral injury, 24 cases (11.54%) were penile injuries and 22 cases (10.58%) presented with injury to the scrotum/testiculus. The site of injury, cause of injuries, related concomitant injuries, diagnostic methods, methods of treatment and outcome were then reviewed. The follow-up information was analyzed and summarized.

## RESULTS

Among the 208 cases, 197 were males and 11 were females, with ages ranging from 11 to 75 years and an average of 37.73 years. Among them, sixty-two cases (29.81%) were kidney injuries, of which 25 cases involved the left kidney, 35 cases had right kidney trauma, and 2 cases manifested bilateral kidney injuries. In addition, two cases also had liver rupture, two cases had splenic rupture, one patient also had a pulmonary contusion and eight cases also had limb fractures. Regarding the cause of injuries, the kidney injuries were grouped into traffic injuries (34 cases, 54.84%), fall-related injuries (25 cases, 40.33%), violence-related injury (1 case, 1.61%), knife injury (1 case, 1.61%), and spontaneous rupture of the giant hydronephrosis (1 case, 1.61%). There were two cases of ureteral injury (0.96%), which were both left ureter injuries caused by maloperation during gynecologic laparoscopy. Bladder injury was observed in six patients (2.88%), among which four cases were extraperitoneal and two cases were intraperitoneal; three bladder-injured patients were combined which also had a pelvic fracture: one case had a confirmed intestinal rupture, and another case had an accompanying perineum wound. These bladder injury cases were divided into three cases of traffic injury (50%), two cases of drunken falls (33.33%), as well as one case of iron stab injury (16.67%), based on the injury causes. Urethral injury occurred in 92 cases (44.23%), among which 20 were bulbous urethral injury (n=72) and 20 involved the membranous urethra.

Additionally, 20 patients with urethral injury also had pelvic fracture, while one case had an intestinal rupture, and 5 patients had contusion and laceration of the scrotum. According to causes, urethral injury was separated into traffic injuries (47 cases, 51.09%), fall injuries (39 cases, 42.39%), and crush injury from machines (6 cases, 6.52%). Male reproductive system injuries were observed in 46 cases (22.12%), among which 24 cases demonstrated penile damage, 14 cases were pure scrotum damages, 8 cases presented with a combined injury to the scrotum and testiculus, and two cases also had urethral injury. According to the causes of injuries, they were grouped into 21 cases of scrotum collision, (45.65%), 15 cases of bending injury to the erect penis (32.62%, 10 cases occurred during sexual intercourse), one case of blast injury (2.17%), and one case of bite injury (2.17%). The interval between disease onset and hospital admission of the 208 patients ranged from 20 min to 4 days, with a median of five hou;rs.

Sixty-two patients with renal injuries were examined by ultrasound and computed tomography (CT) examination. Renal injuries were classified into five grades based on the American Association for the Surgery of Trauma Renal Injury Grading System (Table-I). There were 13 cases of Grade I injuries, 21 cases of Grade II injuries, 13 cases of Grade III injuries, 13 cases of Grade IV injuries, and 2 cases of Grade V injuries.

**Table-I:** Renal injuries were classified into five grades based on the American Association for the Surgery of Trauma Renal Injury Grading System.

Grade	Definition
I	Contusion or nonenlarging subcapsular hematoma, but no laceration
II	nonexpanding perinephric in the limited retroperitoneal space
III	laceration > 1 cm, with no evidence of urine extravasation
IV	laceration extending to the renal parenchaya and renal pelvis, with segmental renal artery and vein injury
V	kidney smashing wound or renal pedicle avulsion

Treatment modalities were selected based on the degree of severity of renal injury (Table-II). Conservative treatment was followed in 45 cases (72.58%), superselective renal arterial chemoembolization was conducted in 9 patients (14.52%), superselective renal arterial chemoembolization following failed conservative management was performed in 9 cases (14.52%), and nephrectomy after a renal probe was executed in 8 subjects (12.90%). Accompanied injuries were also managed. A total of 21 patients (33.87%) received blood transfusion treatment.

**Table-II:** Composition of Patients with Different Grades of Renal Trauma who Received Different Treatments.

Grade	Conservative Treatment (%)	Superselective Arterial Embolization (%)	Nephrectomy (%)	Total
I	13 (100)	0 (0)	0 (0)	13
II	21 (100)	0 (0)	0 (0)	21
III	10 (76.92)	3 (23.08)	0 (0)	13
IV	1 (7.70)	6 (46.15)	6 (46.15)	13
V	0 (0)	0 (0)	2 (100)	2

Intravenous pyelogram (IVP) was conducted in two patients with ureteral injury and the results indicated that the injuries were all located at the left ureteral lower segment in the vicinity of the bladder entrance. A ureteral stent was successfully placed in one patient under a cystoscope, and ureteral reimplantation after a failed catheter insertion was performed in another patient. Neither of these two cases received a blood transfusion.

The diagnosis of bladder injury was confirmed in six patients using a waterflood susceptibility test combined with CT scans. All these patients underwent laparotomy, and the bladder suture was done after identification of the rupture sites. Injuries to other parts were also appropriately managed, and a total of five patients (83.33%) underwent blood transfusion treatment.

Urethral catheterization was performed in 22 cases of bulbous straddle injury resulting from a fall or a car accident, among which 5 attempts succeeded (6.94%). Those cases with unsuccessful catheterization further underwent microscopic examination, by which the lesion sites were clearly observed. Ureterorenoscopic realignment was successfully executed in 35 cases (48.61%). Thirty-two patients who underwent a failed attempt at catheter placement were transferred to an open operation, among which 28 cases (38.89%) were treated with urethral anastomosis, and another 4 cases (5.56%) were managed with urethroplasty. Membranous urethra traumas were identified in 20 cases based on CT images indicative of pelvic fractures, and the patients were then treated with urethral catheterization. Nevertheless, the treatment only succeeded in one case (5%). Among the 19 subjects who underwent a failed attempt at catheter placement, 18 (90%) were managed with urethral realignment, and 1 (5%) was treated with end-to-end anastomosis of the urethra. Other combined injuries were appropriately managed (pelvic fractures were treated with nonoperative modalities), and a total of 24 patients (25%) received blood transfusion treatment.

Of the 24 cases with penile injuries, one underwent conservative treatment (4.17%), 8 were treated with debridement and suture ligation (33.33%), 15 were managed with suture repair of the penis white membrane (62.50%). Furthermore, two patients with penile injury combined with urethral injury were treated with urethroplasty and end-to-end anastomosis of the urethra, respectively. Among the 22 cases presented with injury to the scrotum/testiculus, 21 of them were diagnosed with scrotum and testiculus injury based on ultrasonic examination. Among them, 8 cases suffered from unilateral testis injury and 13 cases were afflicted with unilateral scrotum injury. The diagnosis was not determined in only one case; thus, scrotal exploration was finally performed and an intact testiculus was observed. Among the 14 patients with unilateral scrotal injury, one case was subjected to conservative treatment (7.14%), and two subjects two underwent scrotal exploration/debridement and suturing (92.86%). For the eight patients who demonstrated testicular injuries, conservative modalities were performed in one case (12.50%), repair of the testiculus was conducted in two cases (25%), and orchiectomy was administered in five patients (62.50%). Three out of the 46 cases (6.52%) received a blood transfusion (7.14%).

No deaths were found in any of the 208 patients, and they were all cured and discharged from hospital. The total length of hospital stay ranged from 1 to 52 days, with an average stay of 9 days. They were followed up for 1–24 months after being discharged from hospital, with a median follow-up period of 12 months. During the follow-up period, normal renal function was observed in 62 patients who suffered previously from renal injury. Furthermore, postoperative IVP and ECT were performed in nine subjects following superselective arterial embolization, and normal renal function in the previously affected kidney was found. There was no hydronephrosis in the two patients who had presented with ureteral injury previously. Six patients with bladder rupture were cured, and normal urination was observed. Of the 92 cases that displayed urethral injury, 29 cases developed urethrostenosis after catheter removal (31.52%). Among them, involvement of the membranous portion was found in 11 cases (accounting for 55% of all membranous portion injuries), and involvement of the bulbous urethra was seen in 18 cases (accounting for 25% of all membranous bulbous injuries); in those patients with urethral injuries, 19 of them were treated with end-to-end anastomosis of the urethra (18 cases were completely cured, and urethrostenosis recurred in one case). Good efficacy was seen in three patients who managed with an internal urethrotomy. Urethral dilatation was conducted periodically to maintain micturition in five subjects, and another two cases underwent permanent bladder catheter retention due to repeated surgical failures. All of the 46 male patients with both urethral injuries and reproductive system trauma recovered well during the follow-up period.

## DISCUSSION

In China, it is generally considered that grade I and II kidney injury are supposed to be treated conservatively and Grade III and IV kidney injury should be treated with surgery as soon as possible. In other countries, conservative treatment is considered as the standard treatment for grades I-III renal trauma.^2^ McGuire *et al.*^6^ summarized the treatment efficacy of 117 patients with high-grade renal trauma (grade III-V) and found that 14 out of 27 cases were successfully treated with conservative modalities (52%). Therefore, they believed that as long as hemodynamic stability was observed, a conservative treatment could be considered despite the severity of the damage. However, the hemogram should be closely monitored and CT scans should be continuously reviewed. Superselective renal arterial chemoembolization is a relatively new and rapidly advancing field. A renal artery angiography for an affected kidney is first performed under an endoscope to display bleeding arterial branches. The microcatheter is further placed into the detected bleeding arterial branches, which is embolized with gelatin sponge particles afterwards, and embolization is then accomplished. Our present research results demonstrated that superselective renal arterial chemoembolization was superior than other treatments. It can not only swiftly and effectively manage hemorrhage occurring in acute renal injury but also maximally protect kidney tissues and restore renal function. For high-grade renal trauma, especially for grade V renal injury, the treatment efficacy of superselective renal arterial chemoembolization was relatively poor. For now, surgical exploration is irreplaceable under life-threatening conditions.

Traumatic ureteral injuries are relatively uncommon and mainly due to iatrogenic injury, among which, 50% of them occur in gynecological laparoscopic surgery and 89% of them are found in the low segment of the ureter.^7^ Ureteral injury can lead to serious consequences. Hove *et al.*^8^ analyzed the clinical data of 136 patients with ureteral injuries and found that 34 patients developed irreversible damage of the affected kidney. The diagnosis, which can be confirmed by IVP site, should be further determined. Once the diagnosis is established, it should be handled in time. When difficult catheterization occurs during the process of ureteral catheterization, open repair should be implemented. Repeated catheter placement is not recommended to avoid aggravating ureteral injury.^9^

The diagnosis of bladder and urethra injury may be missed under plain CT scans.^10^ The waterflood susceptibility test, a preferred method for diagnosing bladder rupture, can accurately determine the fracture site and urinary extravasation with a sensitivity of more than 90%. Recently, some scholars have pointed out that CT cystography can also reveal III damages in other parts at the same time; thus, its use has exceeded traditional cystography and become the best diagnostic method.^11^ The European Association of Urology Guide,^2^ points out that most extraperitoneal ruptures of the bladder caused by blunt injury can be treated conservatively via indwelling catheterization, even in the presence of extensive retroperitoneal or scrotal urinary extravasation III. If bladder neck injury or spiculum penetration into the bladder wall occurs, surgical treatment is required. Intraperitoneal bladder ruptures or bladder open wounds are in urgent need of surgical exploration and repair.

The male urethra is more vulnerable to injury due to its anatomical position. At first, the guide wire is placed into the injury site under a cystoscope, and a catheter is placed along the guide wire afterwards. This procedure is called urethral realignment. It is commonly applied in bulbous urethral injury after a failed catheterization attempt. The emergency management of anterior and posterior urethral injury is quite different. In our group, 48.61% of bulbous urethral injuries were treated with ureterorenoscopic realignment, and 38.89% were managed with end-to-end anastomotic urethroplasty; 90% of the membranous urethral injuries were handled with realignment. Urethral stricture is the major complication of urethral injury with an observed incidence of 31.52%, and its incidence is greater in membranous urethral injuries than in bulbous urethral injuries (55% versus 25%). Recently, Huang *et al*.^12^ demonstrated that ureterorenoscopic realignment had a lower incidence of posterior urethral stricture and formation of a pseudo urethra compared with traditional realignment.

Penile injury can arise from blunt force or an open wound. We noticed that fracture of an erect penis (blunt injury) accounted for the majority of the 24 penile trauma cases, and most of them occurred during sexual intercourse with a reported incidence rate of nearly 60%.^13^ Most of the patients did not need an extra imaging examination before surgical exploration and repair.^14^ If penile injury is accompanied by urethral injury, then a urethral repair is needed. For mild and shallow laceration of a penile open wound, debridement and suturing are recommended. Nevertheless, if the wound is deep and its severity is difficult to determine, or if penial rupture occurs, then surgical exploration and repair are required.

Conservative treatment can be performed only when testicular injury can be ruled out and limited hematoma is observed by B-mode ultrasound. If the severity of the testicular injury is not determined or hematoma is relatively large, then surgical exploration and repair should be implemented. In the treatment of testicular injury, the testiculus should be preserved as much as possible. In the present research, 50% of the damaged testicles were preserved, which was consistent with the results of Hudak *et al*.^15^ For simple scrotal hematoma, the hematoma should be totally removed. The treatment principle of a scrotal open wound is similar to that of a penile open wound.

## CONCLUSION

Urethra and kidney injuries are the most common acute urogenital system traumas, followed by the male reproductive system and bladder injuries, while ureteral injury has the lowest incidence. CT can accurately reflect the severity of kidney injury, based on which an appropriate clinical treatment can be implemented. Superselective arterial embolization can effectively cease bleeding and maximally protect renal function, which is a minimally invasive and safe interventional technique. Ureterorenoscopic realignment is an easily operative and minimally invasive technique in the treatment of urethral injuries. Therefore, its efficacy in urethral injuries is worth exploring. With advances in medical technologies, the treatment modalities for urogenital system damage have become less invasive and minimally invasive procedures should widely be used in acute urogenital trauma in future.
